# Drug-resistant tuberculosis in Zhejiang Province, China: an updated analysis of time trends, 1999–2013

**DOI:** 10.1080/16549716.2017.1293925

**Published:** 2017-06-04

**Authors:** Beibei Wu, Le Zhang, Zhengwei Liu, Haibo He, Aizhen Pan, Fei Wang, Mingwu Zhang, Bin Chen, Zuhong Lu, Songhua Chen, Xiaomeng Wang

**Affiliations:** ^a^ Department of Tuberculosis Control and Prevention, Zhejiang Provincial Center for Disease Control and Prevention, P. R. China; ^b^ Department of Epidemiology and Biostatistics, School of Public Health, Peking University, Beijing, P. R. China; ^c^ Department of Biomedical Engineering, College of Engineering, Peking University, Beijing, P. R. China

**Keywords:** surveillance, multidrug resistant TB, second-line drug resistance

## Abstract

**Background**: Multidrug-resistant tuberculosis (MDR-TB) and extensively drug-resistant tuberculosis (XDR-TB) hinder the progress of TB control.

**Objective**: To track the trend of drug-resistant tuberculosis (DR-TB) prevalence in Zhejiang Province from 1999 to 2013, and identify risk factors of resistance to second-line drugs among MDR-TB patients.

**Design**: Four DR-TB surveys had been done in Zhejiang Province in 1999, 2004, 2008 and 2013 through questionnaires, in which demographic and epidemiological items were included. After questionnaires, drug susceptibility testing (DST) targeted at four first-line drugs was done for all TB patients and DST targeted at six second-line drugs (only in 2008 and 2013) for MDR-TB patients. The drug resistance trend over time was analyzed using the Cochran–Armitage test. The factors associated with resistance to second-line drugs among MDR-TB patients were examined by a multivariate logistic regression model.

**Results**: Of 936 patients enrolled, 27 (3.21%) and 20 (21.28%) MDR-TB cases were registered as new and previously treated cases, respectively. MDR-TB showed a decreasing trend (Z = −3.31, *p* < 0.01) while resistance to any first-line drugs showed an increasing trend (Z = 5.22, *p* < 0.001), from 1999 to 2013. The highest resistance rate was shown to ofloxacin among MDR-TB patients both in 2008 (28.8%) and in 2013 (27.7%), while resistance to para-aminosalicylate decreased significantly (Z = −2.06, *p* = 0.04) between 2008 and 2013. MDR-TB patients aged 45–65 years (OR = 5.00, *p* = 0.02) were more likely to be resistant to any second-line drugs.

**Conclusions**: DR-TB including MDR-TB remains a major public health problem in Zhejiang Province. Further efforts on MDR-TB control should be conducted to hinder drug resistance, including critical clinical use of anti-TB antibiotics and preventing transmission.

## Background

The prevalence of multidrug-resistant tuberculosis (MDR-TB) in China was estimated to be 1.7 and 1.3 times of the global average among new and previously treated patients respectively in 2014 [1,2]. The emergence of drug-resistant tuberculosis (DR-TB), especially MDR-TB and extensively drug-resistant tuberculosis (XDR-TB), has lowered the success rate of anti-TB treatment and therefore kept primary cases of MDR and XDR-TB in China sustainable [2–4]. Monitoring DR-TB to adjust to treatment regimens and preventive strategies is vital for reducing TB burden [5,6]. However, due to the high cost of drug-susceptibility testing (DST), monitoring drug resistance time trends has not yet been set up at the national level in China, and DST is not compulsory before initiating treatment on TB patients. Usually DST is to be carried out only when the patient does not show a clinical effect after two or five months of treatment [7,8].

Zhejiang Province is one of the most developed provinces in China and has constructed a routine monitoring system for drug resistance since the World Health Organization (WHO) integrated it into its surveillance network of DR-TB in 1999. According to the code of the WHO, Zhejiang conducted four cross-sectional surveys on drug resistance in 1999, 2004, 2008 and 2013. DST for first-line drugs (isoniazid [INH], rifampin [RFP], ethambutol [EMB] and streptomycin [SM]) was implemented in each survey but DST for six second-line drugs (ofloxacin [OFX], kanamycin [KM], amikacin [AM], capreomycin [CPM], cycloserine [CS] and para-aminosalicylic acid [PAS]) among MDR-TB was only available in 2008 and 2013.

Our previous study showed that the prevalence of MDR-TB decreased from 8.6% in 1999 to 6.0% in 2008 and INH resistance was related to RFP resistance [9]. This study tried to continually explore the time trend of MDR-TB and identify the profile of second-line drug resistance among MDR-TB between 2008 and 2013, and to determine the factors associated with resistance to second-line drugs, knowledge of which is still unavailable in some high drug resistance burden countries [1].

## Method

### Study and subject

Our study aimed to evaluate levels and trends of DR-TB in Zhejiang Province. All 30 counties surveyed in 1999 were included in the fourth survey in 2013 except for one county. We would need 784 cases (i.e. 1.96^2^_0.05_0.5(1 − 0.5)/(0.07/2)^2^ = 784) to achieve 95% precision and a margin of error of 7%, assuming no prior knowledge of prevalence of drug resistance, to measure prevalence of drug resistance across Zhejiang Province in each survey. Assuming that > 10% of samples would be lost, we sought to obtain 900 cases. We randomly selected 30 TB treatment centers in 30 counties among 90 centers in Zhejiang Province and planned to recruit 30 sputum smear-positive patients in each center. Details can be found in our previous study (Jia) [9].

Patients who came to healthcare facilities and diagnosed as sputum smear-positive TB were eligible for inclusion and then a section of a questionnaire was provided. For patients aged under 14 or with intellectual disabilities, the questionnaire was filled out by their guardians and/or parents together. The enrollment was sequentially implemented until 30 new patients were enrolled in each site.

New cases were defined as cases who had never previously had treatment for TB, or had taken anti-TB drugs for less than one month. Previously treated cases were defined as cases who had received one month or more of anti-TB drugs in the past, and they were further classified by the outcome of their most recent course of treatment [10]. Information about patient registration group was acquired directly through questioning by clinicians, and data entry was done by clinicians and disease control personnel independently that day. To avoid misclassification bias, project personnel from Zhejiang Provincial Center for Disease Control and Prevention (CDC) checked the information every week. If inconsistent results occurred, information was checked by the three people together. Telephone survey, household survey and asking family members were possible methods. The questionnaire included items on baseline information like age, gender, education and family income and clinical information like treatment history, symptoms and diabetes status of the patients. Informed consent was obtained from all patients.

### Laboratory methods

Sputum smear microscopy was performed directly at the TB bacterium laboratory in each county, where direct acid-fast bacillus (AFB) smear microscopy and solid LJ culture were used. The identification of *Tuberculosis* and DST was performed at the provincial reference laboratory, DST was carried out on LJ media by the proportion method and the same method was used for all drugs, and results were compared with those from the standardized strains. Drug concentrations for RMP, INH, EMB, SM, OFX, KM, AM, CPM, PAS and CS were 40.0, 0.2, 2.0, 4.0, 2.0, 30.0, 40.0, 40.0, 1.0 and 40.0 μg/ml respectively. Quality of the provincial reference laboratory was ensured by the Republic of Korea Supranational Reference Laboratory by determining the final resistance pattern, and was evaluated annually by the national reference laboratory of China. Details of the design and methods have been described previously [9].

### Statistical analysis

The drug resistance trend over time was analyzed using the Cochran–Armitage test for trend. The factors associated with MDR-TB were examined by a multilevel multivariate logistic regression model, in which the region was fitted as 3-level to detect spatial aggregation, year of survey as 2-level and each individual as 1-level. A multivariate logistic regression model was used to identify independent factors associated with resistance to any second-line drugs among MDR-TB patients, by estimating OR and 95% CI for each factor. We took *p*-values < 0.05 as significant, and < 0.001 as highly significant. The Cochran–Armitage trend test and logistic regression were done using SAS 9.1 (SAS, Inc., Cary, NC). Multilevel multivariate logistic regression analysis was done with MLwiN 2.02 (Multilevel Models Project Institute of Education).

## Results

A total of 1010 sputum-positive patients were included in the fourth TB drug resistance surveillance of Zhejiang Province. The survey was performed from February 2013 to August 2014. Of these, 23 smear samples showed negative results (no mycobacterial growth) and 51 strains were detected as non-TB mycobacterium. The final study population consisted of 936 individuals.

There were no statistically significant demographic differences between non-MDR-TB and MDR-TB patients (Table 1). Patients of the two groups differed in terms of treatment category: 74 (8.3%) non-MDR-TB patients were registered as previously treated, versus 20 (42.6%) of the MDR-TB patients (*p* < 0.001).

**Table 1.** Demographic and clinical characteristics among non-MDR-TB patients and MDR-TB patients in Zhejiang, China, 2013.

Characteristics	Total (n = 936)	Non-MDR-TB (n = 889)	MDR-TB (n = 47)	*p*-value
**Demographic characteristics**				
Age group				0.315
< 25 yrs	172 (18.4)	161 (18.1)	11 (23.4)	
25–45 yrs	326 (34.8)	315 (35.4)	11 (23.4)	
45–65 yrs	240 (25.6)	225 (25.3)	15 (31.9)	
≥ 65 yrs	198 (21.2)	188 (21.1)	10 (21.3)	
Sex				0.87
Female	280 (29.9)	267 (30.0)	13 (27.7)	
Male	656 (70.1)	622 (70.0)	34 (72.3)	
Residence				0.377
Urban	218 (23.3)	210 (23.6)	8 (17.0)	
Rural	718 (76.7)	679 (76.4)	39 (83.0)	
Education				0.231
None	205 (21.9)	196 (22.0)	9 (19.1)	
Primary school	276 (29.5)	256 (28.8)	20 (42.6)	
Junior middle school	290 (31.0)	281 (31.6)	9 (19.1)	
Senior high school	122 (13.0)	115 (12.9)	7 (14.9)	
University or college	43 (4.6)	41 (4.6)	2 (4.3)	
Health insurance				0.094
Yes	748 (79.9)	715 (80.4)	33 (70.2)	
No	188 (20.1)	174 (19.6)	14 (29.8)	
Family income per year				0.416
< 50 T RMB	542 (57.9)	510 (57.4)	32 (68.1)	
50–100 T RMB	229 (24.5)	218 (24.5)	11 (23.4)	
≥ 100 T RMB	128 (13.7)	125 (14.1)	3 (6.4)	
Unknown	37 (4.0)	36 (4.0)	1 (2.1)	
**Clinical characteristics**				
Treatment category				< 0.001
New patients	842 (90.0)	815 (91.7)	27 (57.4)	
Previously treated patients	94 (10.0)	74 (8.3)	20 (42.6)	
Family members with TB				
Yes	63 (6.7)	60 (6.7)	3 (6.4)	
No	873 (93.3)	829 (93.3)	44 (93.6)	
Symptoms				0.086
Yes	902 (96.4)	859 (96.6)	43 (91.5)	
No	34 (3.6)	30 (3.4)	4 (8.5)	
Diabetes				0.708
Yes	76 (8.1)	71 (8.0)	5 (10.6)	
No	838 (89.5)	797 (89.7)	41 (87.2)	
Unknown	22 (2.4)	21 (2.4)	1 (2.1)	
Hepatitis B				0.46
Yes	26 (2.8)	24 (2.7)	2 (4.3)	
No	881 (94.1)	838 (94.3)	43 (91.5)	
Unknown	29 (3.1)	27 (3.0)	2 (4.3)	
No. of prior treatment episodes			< 0.001	
0	837 (89.4)	810 (91.1)	27 (57.4)	
1	80 (8.5)	65 (7.3)	15 (31.9)	
2	15 (1.6)	12 (1.3)	3 (6.4)	
3	2 (0.2)	1 (0.1)	1 (2.1)	
4	2 (0.2)	1 (0.1)	1 (2.1)	

Notes: T, thousands; RMB, official currency of the People’s Republic of China; MDR-TB, multidrug-resistant tuberculosis.

Factors associated with resistance to MDR-TB are shown in Table 2. Previous treatments was the strongest predictor for MDR-TB (OR = 10.91, 95% CI 9.41–12.65) compared with new TB patients.

**Table 2.** Multilevel multivariate analysis on factors associated with MDR-TB in Zhejiang Province, China, 1999, 2004, 2008 and 2013.

	Total	Adjusted OR (95% CI)	*p*-value
Fixed effect			
Constant	–	0.04 (0.03,0.05)	<0.001
Age group			
<25 yrs	498 (12.6)	Ref	Ref
25-45 yrs	1503 (38.2)	0.91 (0.71,1.16)	0.692
45-65 yrs	1092 (27.7)	0.85 (0.65,1.10)	0.519
≥65 yrs	844 (21.4)	0.63 (0.47,0.83)	0.095
Sex			
Female	1151 (29.2)	Ref	Ref
Male	2786 (70.8)	0.90 (0.77,1.06)	0.526
Treatment category			
New case	3398 (86.3)	Ref	Ref
Previously treated case	539 (13.7)	10.91 (9.41,12.65)	<0.001
Random effect			
Level 3 – region	–	1.27 (1.11,1.45)	0.3
Level 2 – year	–	1.10 (1.00,1.22)	0.068
Level 1 – individual	–	–	–

Notes: OR, odds ratio; MDR-TB, multidrug-resistant tuberculosis.

### Trend of first-line drugs resistance

**Figure 1.**
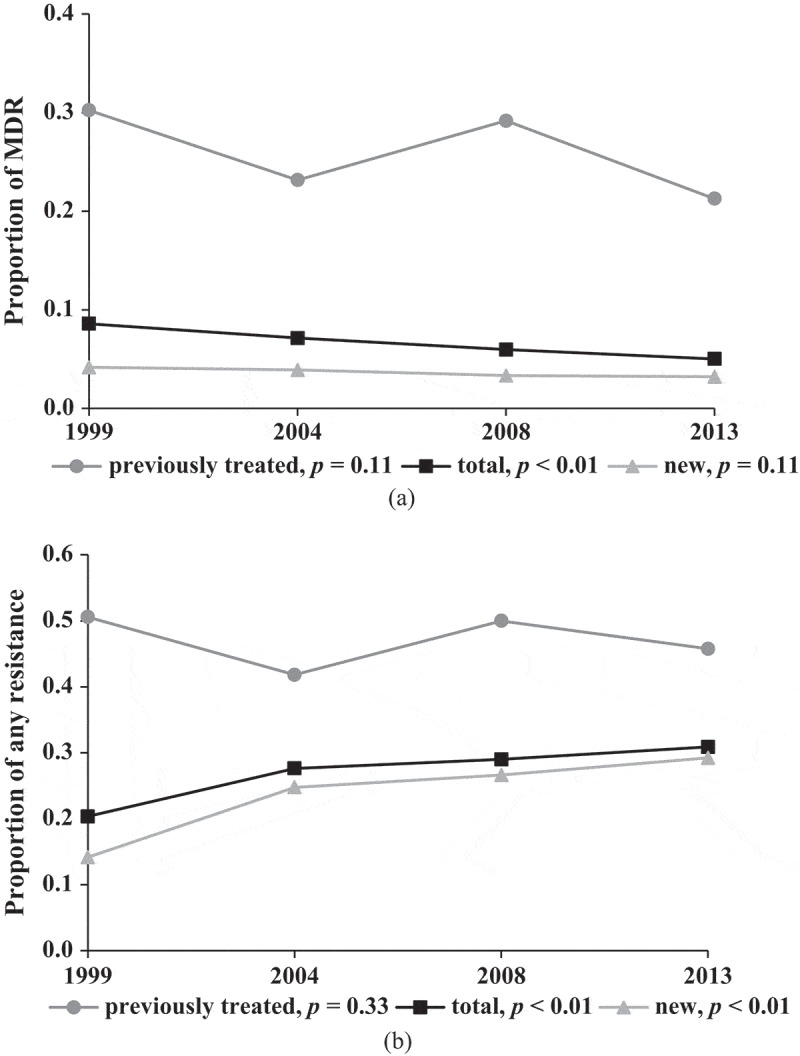
Trends of MDR and resistance to any first-line drugs, 1999–2013. (a) Trends in MDR-TB among new, previously treated and all patients, 1999–2013. (b) Trends in resistance to any first-line drugs among new, previously treated and all patients, 1999–2013.

Figure 1 showed a significant decrease (from 8.6% to 5.0%) of proportion of MDR-TB (Z = −3.31, *p* < 0.01) and an increase (from 20.3% to 30.9%) of proportion resistant to any first-line drugs (Z = 5.22, *p* < 0.001) in the period of 1999–2013. However, the proportions remained steady from 2008 to 2013 for both MDR-TB (Z = −0.90, *p *= 0.37) and resistance to any first-line drugs (Z = 0.89, *p* = 0.37). For new TB patients, proportion of MDR-TB declined consistently without significance (Z = −1.19, *p* = 0.12), while proportion of resistance to any first-line drugs increased sharply from 14.15% in 1999 to 29.22% in 2013 (Z = 7.17, *p* < 0.001). By contrast, those figures for previously treated patients fluctuated over the span of 15 years. In 2013, 29.2% of new patients and 45.7% of previously treated patients were resistant to at least one first-line drug. The proportions of resistance to EMB and SM in 1999–2013 are described in Table S1.

### Resistance to second-line drugs among MDR-TB cases

A total of 52 and 47 MDR strains were tested for susceptibility to six second-line drugs in 2008 and 2013 respectively. Of these, 27 (51.9%) and 17 (36.2%) strains showed resistance to at least one second-line drug with no significant difference (Table 3). The rate of resistance to PAS decreased significantly from 32.7% to 14.9% (Z = −2.06, *p* = 0.04) from 2008 to 2013. Of all the six second-line drugs, OFX showed the highest resistance rate of 28.8% in 2008 and 27.7% in 2013. The lowest-resistance drug was identified as CS (5.8%) in 2008, and AM (4.3%) and CPM (4.3%) in 2013. Factors associated with resistance to second-line drugs are shown in Table 4. Patients aged 45–65 were more likely to be resistant to any second-line drugs compared with patients under 25 (OR = 5.00; 95% CI: 1.31–19.07; *p* < 0.05). Previously treated patients were not at a higher risk of being resistant to any second-line drugs compared with new patients (OR = 1.01; 95% CI: 0.41–2.49; *p* = 0.98). Factors like sex, occupation and year were also not associated with resistance to any second-line drugs.

**Table 3.** Resistance to second-line drugs among MDR strains, 2008 and 2013.

	Resistant no. (%)	
Drug, year	2008	2013
Total MDR no.	52	47
Ofloxacin	15 (28.8)	13 (27.7)
Para-aminosalicylate	17 (32.7)	7 (14.9)
Cycloserine	3 (5.8)	5 (10.6)
Aminoglycosides	7 (13.5)	4 (8.5)
Kanamycin	5 (9.6)	4 (8.5)
Amikacin	4 (7.7)	2 (4.3)
Capreomycin	6 (11.5)	2 (4.3)
Any resistance	27 (51.9)	17 (36.2)

Note: The results of second-line drugs susceptibility testing were available for 52 patients among 56 cases of MDR-TB in 2008; MDR-TB, multidrug-resistant tuberculosis.

**Table 4.** Multivariate analysis on factors associated with resistance to any second-line drugs among MDR-TB patients.

Predictors	Resistant no. (%)	Susceptible no. (%)	OR (95% C.I)	*p*-value
Age group				
< 24 yrs	4 (25.0)	12 (75.0)	Ref	
25–45 yrs	12 (36.4)	21 (63.6)	1.71 (4.45–6.52)	0.43
45–65 yrs	20 (62.5)	12 (37.5)	5.00 (1.31–19.07)	< 0.05
≥ 65 yrs	8 (44.4)	10 (55.6)	2.40 (0.55–10.38)	0.24
Sex				
Female	9 (32.1)	19 (67.9)	Ref	
Male	35 (49.3)	36 (50.7)	1.98 (1.74–5.27)	0.17
Occupation				
Other	27 (49.1)	28 (50.9)	Ref	
Farmer	17 (38.6)	27 (61.4)	1.31 (6.51–3.42)	0.57
Treatment history				
New	23 (42.6)	31 (57.4)	Ref	
Previously treated	21 (46.7)	24 (53.3)	1.01 (0.41–2.49)	0.98
Year				
2008	27 (51.9)	25 (48.1)	Ref	
2013	17 (36.2)	30 (63.8)	0.50 (6.21–1.23)	0.12

Notes: OR, odds ratio; MDR-TB, multidrug-resistant tuberculosis.

## Discussion

The primary finding in our updated analysis presented an insignificant decrease of proportion of MDR-TB from 6.0% to 5.0% between 2008 and 2013 in Zhejiang, as well as in both new cases (3.3% to 3.2%) and previously treated cases (29.1% to 21.3%). The decline (15.9%, from 5.97% to 5.02%) in 2008–2013 was slightly slower than that in 1999–2004 (16.9%; from 8.59% to 7.14%) and in 2004–2008 (16.4%; from 7.14% to 5.97%). This result was mainly attributable to increased resistance to SM, which had been used for many years as the first antibiotic for TB treatment [11]. However, mono-resistance to INH, RFP and EMB did not show significant increase from 1999 to 2013 (Table S1). China has conducted a series of strategies on TB prevention and control since 1993 [12]. These works were supported by both external funding initiatives, from the World Bank during 1993–2008 and the Global Fund to Fight AIDS, Tuberculosis and Malaria during 2003–2013, and the increasing domestic expenditure launched by the Chinese government to reinvigorate Directly Observed Treatment, Short Course (DOTS) since 2002, targeted to improve TB control in poorer areas in China [13]. This battle undoubtedly contributed to the control of MDR-TB in 1993–2013, but it was difficult to eradicate the endemic only by these measures, particularly under the current circumstances where its prevalence is still higher than the global average rate of new (3.3%) and previously treated (20%) MDR-TB patients [14]. Zhejiang’s gross domestic product (GDP) ranked fourth consistently among all 31 provinces and municipalities in China over the 15 years, and the Province has conducted local surveillance every five years since 1999, a long time earlier than the first national survey of DR-TB in 2007. In 2013, the rates of new (3.2%) and previously treated (21.3%) MDR-TB patients were lower than those of the national averages (5.7% and 26%), but close to the global averages (3.5% and 20.5%). By 2002, DOTS coverage achieved 100% in Zhejiang. Implementing of DOTS achieved a high cure rate by reducing the inadequately treated cases, thus contributing to the decrease of the MDR-TB level. Though diagnostic capacity improved, rapid diagnostic methods are still not in use. The slow decrease also indicated that MDR-TB remains a challenge in Zhejiang Province. Careful management of TB treatment and applying new diagnostic methods are still needed in China.

Previous TB treatment was not surprisingly a predictor for MDR-TB, which was the same case in previous studies [15–18]. However, our study further indicated that MDR-TB was also different by subcategories of previously treated cases. In the fourth survey, the rate of MDR-TB was 50%, 18.3% and 0% in the category groups of the treatment after failure patients, relapse patients and treatment after loss to follow-up patients, respectively (Figure S1). This differed from the WHO surveillance data which found the level of MDR to be 49% in treatment failure patients and 32% in relapse and default patients [19]. Patients registered in the group of treatment after loss to follow-up were not likely to gain resistance in Zhejiang Province, compared to treatment failure patients and relapse patients. These findings can guide the effective use of standard treatment regimens for different patient groups.

Resistance to six second-line drugs has been tested in 2013 and unoptimistic results have been disclosed. Five of the six second-line drugs did not present a significant decline between 2008 and 2013. Resistance to OFX among MDR-TB patients ranked second in 2008 (28.9%) and first in 2013 (27.7%), with figures higher than those of the Chinese national average (25.4%), Thailand (15.0%), South Africa (4.8%) and India (9.0%) [2,20–22]. OFX is a broad-spectrum antibiotic which is usually used in clinical treatment for many other infections [23]. A study on antibiotic use in primary health care settings revealed that 57% of prescriptions for outpatients were proper in Zhejiang Province [24]. This strongly indicated that to achieve better control of DR-TB in China, more attention must be paid to proper antibiotic use in clinics. Resistance to PAS decreased significantly over five years from 32.7% to 14.9%, but was still much higher than the national average of 3.2% [2]. PAS was reported to be of great efficacy for MDR-TB treatment in a randomized, controlled, blinded assessment in Sweden and was recorded to be advantageous in prevention of resistance [25]. The higher resistance rate in Zhejiang Province suggests that use of PAS should be brought under careful management.

The patients aged 45–65 had a higher risk of resistance to second-line drugs (OR = 5.00; 95% CI: 1.31–19.07) compared with patients under 25. This may be attributable to the side effects of first-line drugs and the economic status of this population. The first-line drugs were reported to be age-related with hepatotoxicity, in addition the second-line drugs are not free for TB treatment in China because of high cost [26,27]. Patients of 45–65 years of age usually enjoy a good economic condition and therefore could afford better treatment regimens composed of second-line drugs [21].

We did not find a higher risk of resistance to second-line drugs among the previously treated patients, which was not in accordance with some studies [28]. Resistance to second-line drugs among MDR-TB in Zhejiang Province may not be attributable to poor treatment adherence or inadequate treatment since coverage of DOTS achieved 100% in 2002 with a high treatment success rate officially reported [29]. One of the possible explanations is the improper use of anti-TB antibiotics in clinics. Another possible reason is the transmission of second-line drug resistant *M. tuberculosis* strains. Gao Qian’s study reported that most Chinese DR-TB patients were primary cases [30].

The current study has limitations. First, we could not detect all potential risk factors because of limited resources and non-availability of information. Potential risk factors like HIV status, diabetes, living conditions and income were not measured. This might have led to inaccurate estimation of their true effects. Further, the surveillance only represented patients diagnosed in CDCs and hospitals; private health sectors were not included. Those not having access to health facilities were also not included in our study, therefore there was bias when it came to representing the community’s situation. Nevertheless, given the little information available about second-line drugs among MDR-TB in China, our work can provide implications for MDR-TB control in similar contexts, and generally give implications of the trend of the TB resistance rate in Zhejiang over a period of 15 years.

## Conclusions

Our findings from monitoring the DR-TB trend against both first- and second-line drugs in Zhejiang Province provided general implications on TB control to regions with similar contexts. The impact of general clinical use of anti-TB antibiotics and transmission of drug-resistant *M. tuberculosis* strains on the prevalence of DR-TB are considerable issues for future studies.

## Paper context

Knowing the trend of the resistance rate of tuberculosis and patient characteristics can help local governments control tuberculosis, but information on those is limited. This study aims to track the time trend of the resistance rate over 15 years in Zhejiang Province, and determine risk factors for multidrug-resistant tuberculosis and resistance to second-line drugs. Careful management of drug-resistant tuberculosis, proper use of antibiotics and applying new diagnostic methods are needed in China.
