# Erratum

**DOI:** 10.1080/16549716.2017.1395149

**Published:** 2017-10-26

Wu B., Zhang, L., Liu Z., He H., Pan A., Wang F., Zhang M., Chen B., Lu Z., Chen S and Wang X. 2017. *Drug-resistant tuberculosis in Zhejiang Province, China: an updated analysis of time trends, 1999-2013*.

http://dx.doi.org/10.1080/16549716.2017.1293925

The following line was omitted from the version originally published online:

Joint first authors, B.W and L.Z, contributed equally to this article.

This information has now been re-instated in the acknowledgements section and indicated as a footnote on the first page.

Taylor and Francis apologizes for this error.

